# Glucose Phosphorylation Is Required for *Mycobacterium tuberculosis* Persistence in Mice

**DOI:** 10.1371/journal.ppat.1003116

**Published:** 2013-01-10

**Authors:** Joeli Marrero, Carolina Trujillo, Kyu Y. Rhee, Sabine Ehrt

**Affiliations:** 1 Department of Microbiology and Immunology, Weill Cornell Medical College, New York, New York, United States of America; 2 Department of Medicine, Weill Cornell Medical College, New York, New York, United States of America; McGill University, Canada

## Abstract

*Mycobacterium tuberculosis* (Mtb) is thought to preferentially rely on fatty acid metabolism to both establish and maintain chronic infections. Its metabolic network, however, allows efficient co-catabolism of multiple carbon substrates. To gain insight into the importance of carbohydrate substrates for Mtb pathogenesis we evaluated the role of glucose phosphorylation, the first reaction in glycolysis. We discovered that Mtb expresses two functional glucokinases. Mtb required the polyphosphate glucokinase PPGK for normal growth on glucose, while its second glucokinase GLKA was dispensable. ^13^C-based metabolomic profiling revealed that both enzymes are capable of incorporating glucose into Mtb's central carbon metabolism, with PPGK serving as dominant glucokinase in wild type (wt) Mtb. When both glucokinase genes, *ppgK* and *glkA*, were deleted from its genome, Mtb was unable to use external glucose as substrate for growth or metabolism. Characterization of the glucokinase mutants in mouse infections demonstrated that glucose phosphorylation is dispensable for establishing infection in mice. Surprisingly, however, the glucokinase double mutant failed to persist normally in lungs, which suggests that Mtb has access to glucose *in vivo* and relies on glucose phosphorylation to survive during chronic mouse infections.

## Author Summary

The development of new drugs targeting *Mycobacterium tuberculosis* (Mtb) will benefit from a better understanding of the mechanisms by which this pathogen establishes and maintains chronic infections. Mtb has to adapt its metabolic needs to the nutritional environment in the host. We investigated the role of glucose phosphorylation and discovered that Mtb expresses two functional glucokinases. Using ^13^C-tracing experiments we demonstrated that both enzymes are competent to incorporate glucose into central carbon metabolism. In agreement with the view that Mtb metabolizes fatty acids to grow *in vivo*, both enzymes were dispensable for Mtb replication in mouse lungs and spleens. Surprisingly, however, the glucokinase double mutant was attenuated during the chronic phase of mouse infections. These studies suggest that Mtb metabolizes glucose *in vivo* and that its survival in chronically infected mice depends on glucose phosphorylation.

## Introduction

*Mycobacterium tuberculosis* (Mtb) is the causative agent of tuberculosis, a disease that killed 1.5 million people in 2010 worldwide [1]. The development of new and effective antimycobacterial drugs hinges on our ability to understand how Mtb establishes and maintains chronic infections. Mtb's metabolic adaptations are central to its pathogenicity and prevailing evidence has strongly implicated fatty acids and lipids for biomass and energy production during infection [2]–[6]. In contrast, the importance of other carbon sources including carbohydrates during *in vivo* growth and persistence of Mtb remains uncertain. Mtb possesses carbohydrate transporters and the enzymes required to metabolize sugars [7], [8], but it is unclear whether it utilizes these sugar metabolism pathways to support growth *in vivo*. Mtb is not thought to encounter a sugar-rich environment inside the host, given that expression of sugar catabolism genes was not induced during infection of macrophages and mice [9], [10]. However, sugar transport appears important in Mtb pathogenesis as mutants with transposon insertions in carbohydrate transport systems were attenuated in mouse spleens [11]. The LpqY-SugA-SugB-SugC carbohydrate transporter was confirmed to be indispensable for normal growth in mouse lungs and spleens [12]. This transporter was shown to be highly specific for uptake of the disaccharide trehalose. Trehalose is not present in mammals, but can be released by Mtb from trehalose-containing cell wall glycolipids and recycled [12]. The intracellular fate of recycled trehalose remains to be identified, and it is unknown which other sugars are metabolized by Mtb to maintain fitness during infection.

We sought to investigate the role of glucose metabolism for Mtb pathogenesis and approached this by studying glucokinase, the enzyme catalyzing the phosphorylation of glucose to produce glucose-6-phosphate. This phosphorylation reaction constitutes the first committed step in glucose metabolism activating glucose for oxidation through glycolysis. The Mtb genome contains *ppgK* (*Rv2702*) coding for a polyphosphate glucokinase [7]. Biochemical characterization showed that PPGK catalyzes the direct phosphorylation of glucose using a broad range of phosphoryl donors with a preference for polyphosphate (PolyP) and has a high affinity for the substrate glucose (Km = 0.28 mM) [13], [14]. We generated a *ppgK* deletion mutant in Mtb H37Rv and found that it was severely attenuated for growth with glucose. The mutant, however, retained the ability to grow slowly with glucose as sole carbon source and to incorporate glucose into central carbon metabolism. This led to the identification of a second glucokinase, GLKA, encoded by *Rv0650*. We demonstrate that both PPGK and GLKA are functional glucokinases that allow Mtb to metabolize glucose. Glucose-6-phosphate production by GLKA was, however, low and not sufficient for normal growth of Mtb on glucose. Thus, Mtb primarily relies on PPGK activity for growth on glucose as the sole carbon source *in vitro*. Mtb missing both PPGK and GLKA failed to persist normally in mouse lungs suggesting that glucose is available to Mtb *in vivo* and that Mtb depends on glucose phosphorylation to survive during chronic mouse infection.

## Results

### Mtb expresses two functional glucokinases but relies predominantly on PPGK for growth on glucose

**Figure 1:**
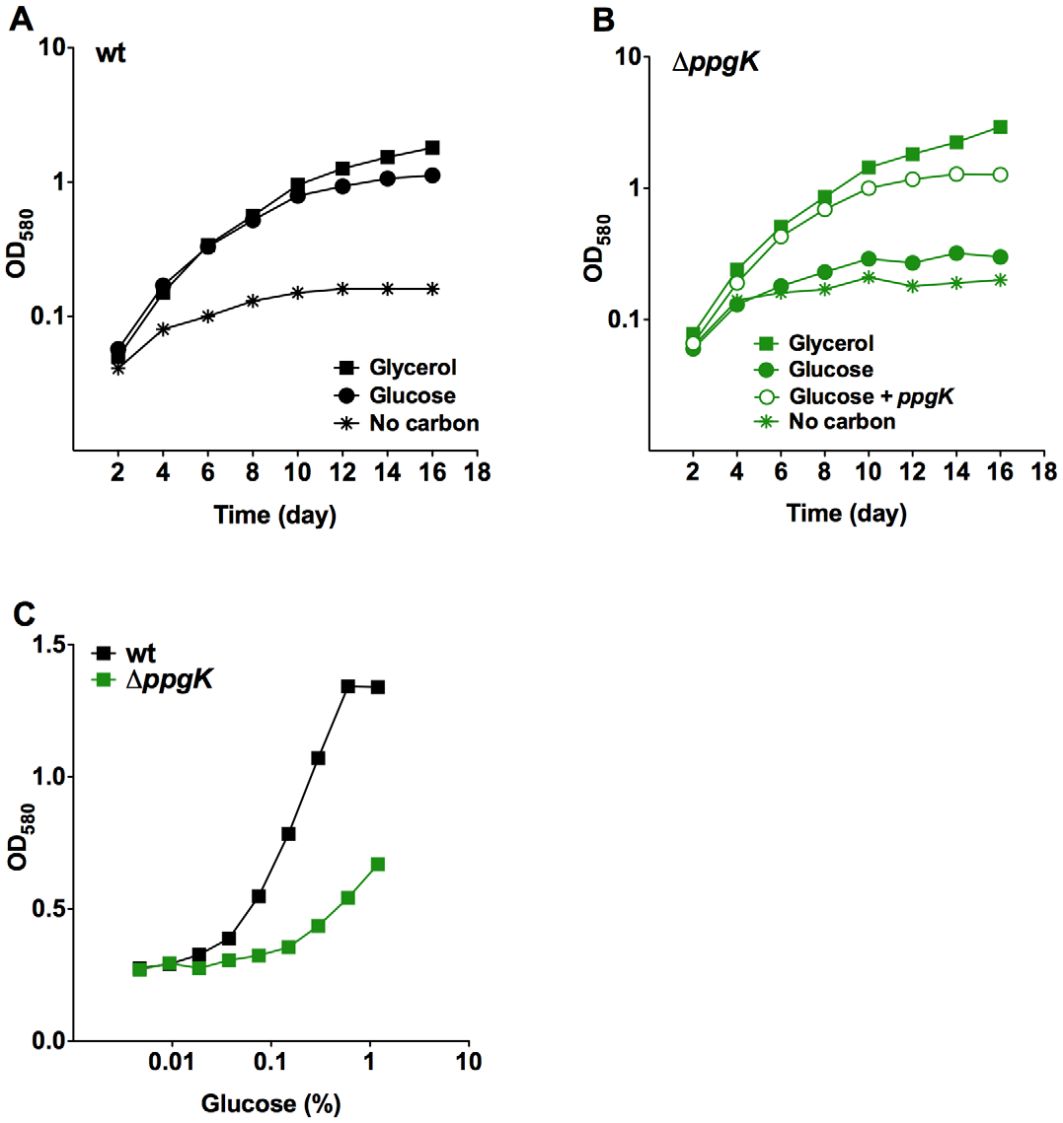
PpgK is required for normal growth with glucose as sole carbon source. Growth of wt Mtb (A) and *ΔppgK* (B) in carbon defined media with 0.4% glycerol (squares), 0.4% glucose (circles) or no carbon (asterisks). Open circles depict growth of the complemented mutant in glucose containing medium. (C) Growth of wt and *ΔppgK* in response to increasing glucose concentration. Data represent two to three independent experiments.

To investigate how PPGK contributes to the metabolism of glucose in Mtb we constructed a *ppgK* deletion mutant (Figure S1A,B) and measured its growth in carbon defined liquid media (Figure 1). Replication of the PPGK mutant (Δ*ppgK*) was severely attenuated compared to wt with glucose as the sole carbon source (Figure 1A,B). The growth defect of Δ*ppgK* in glucose containing medium was restored when a copy of *ppgK* was reintroduced into the genome of the mutant (Figure 1B). We noticed that Δ*ppgK* displayed modest, yet reproducible, replication in glucose containing liquid medium above the no-carbon control. Growth of Δ*ppgK* was also responsive to increasing glucose concentrations in the culture medium (Figure 1C) suggesting that a redundant enzyme mediated some glucose phosphorylation in the absence of PPGK.

**Figure 2:**
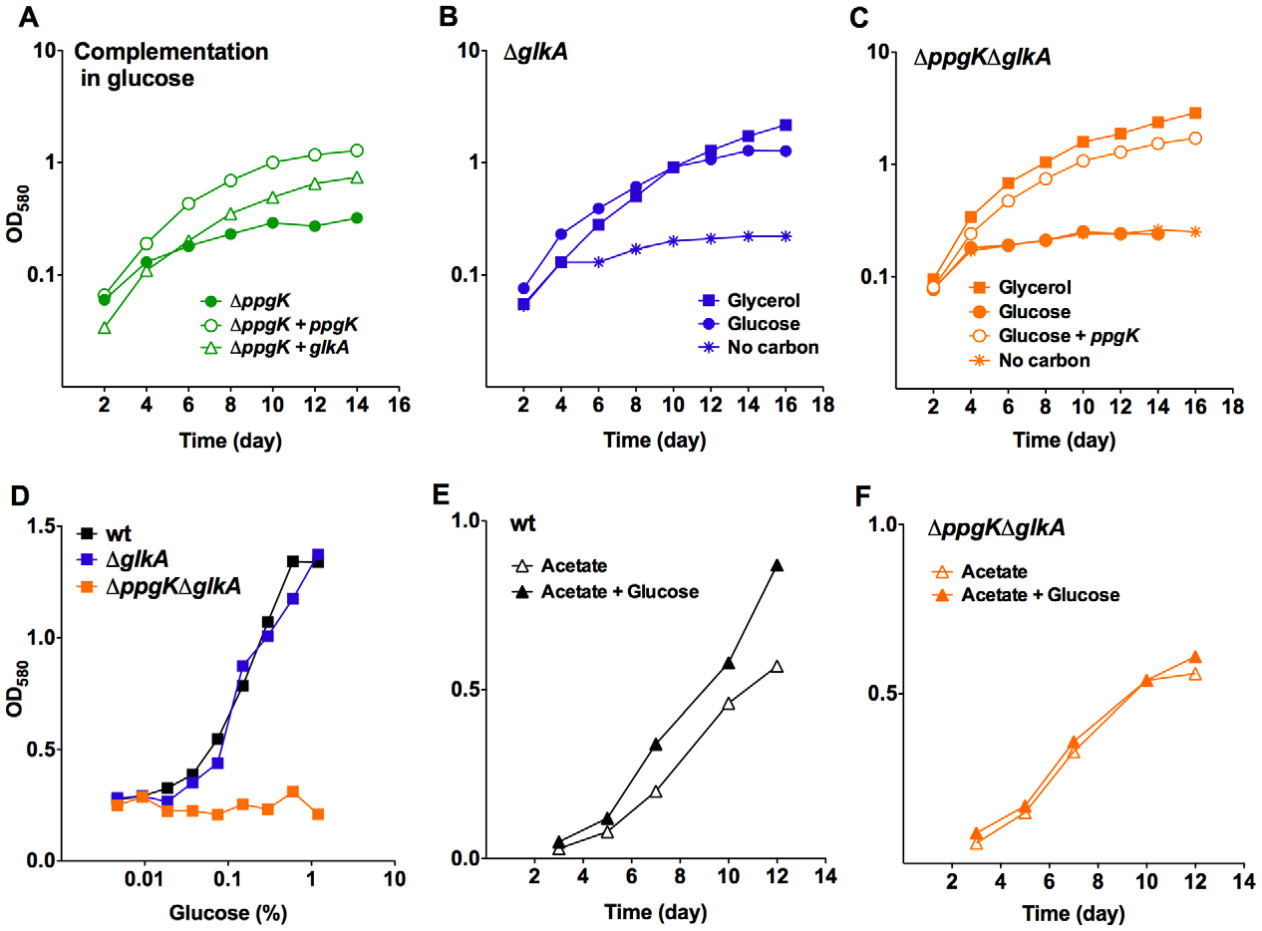
Rv0650 (GLKA) can mediate growth on glucose. (A) Growth of *ΔppgK* and *ΔppgK* complemented with *ppgK* on an integrative plasmid or with *glkA* on an episomal plasmid in carbon defined media with 0.4% glucose. (B, C) Growth of *ΔglkA* (B) and *ΔppgKΔglkA* (C) in carbon defined media with 0.4% glycerol (squares), 0.4% glucose (circles) or no carbon (asterisks). Open circles depict growth of the *ΔppgKΔglkA* mutant complemented with *ppgK* in glucose containing medium. (D) Glucose dose response in liquid media of wt, *ΔglkA* and *ΔppgK ΔglkA*. (E, F) Growth of wt (E) and *ΔppgKΔglkA* (F) in carbon defined media with 0.2% acetate and 0.2% acetate+0.2% glucose. Data represent two to three independent experiments.

The Mtb genome contains a second possible sugar kinase gene, *Rv0650* [7]. Rv0650's amino acid sequence is 71% identical to the GLKA homologue from *Mycobacterium smegmatis*, which was shown to phosphorylate glucose using ATP as the phosphoryl donor [15], but shares only 22% sequence identity with PPGK (Figure S2). To test whether Rv0650 can compensate for the lack of PPGK we expressed the gene from a multicopy plasmid using a strong promoter. This complemented the growth defect of the *ppgK* mutant (Figure 2A) suggesting that *Rv0650* encodes a functional glucokinase that can phosphorylate glucose to support Mtb growth. Based on protein sequence homology to the *M. smegmatis* GLKA and the functional analysis presented in this study we have named the *Rv0650* encoded protein GLKA. In wt Mtb *glkA* transcript levels were similar to those of *ppgk* (Figure S3), suggesting that a difference in mRNA expression does not explain why GLKA could not compensate for the lack of PPGK. We generated a *glkA* deletion mutant and *ppgK/glkA* double deletion mutant (Figure S1C,D). Growth of Δ*glkA* was indistinguishable from that of wt independently of the carbon source (Figure 2B,D) demonstrating that PPGK is sufficient for Mtb to utilize glucose as carbon source for growth.

Growth on glucose as sole carbon source was, however, completely abolished when both glucokinase orthologues *ppgK* and *glkA* were deleted from the Mtb genome (Figure 2C) and Δ*ppgK*Δ*glkA* did not respond to increasing glucose concentration (Figure 2D). Expression of *ppgK* from an integrative plasmid using its putative native promoter restored replication (Figure 2C). As expected, growth of Δ*ppgK*Δ*glkA* on carbon sources generating glucose-6-phosphate through gluconeogenesis was similar to that of wt Mtb (Figure 2E,F and data not shown). Addition of glucose increased the replication rate of wt Mtb on acetate as previously reported [16] (Figure 2E) but did not affect replication of Δ*ppgK*Δ*glkA* in acetate containing medium (Figure 2F). Together, these data indicate that Mtb expresses two functional glucokinases, but relies primarily on PPGK to produce energy and biomass from glucose. The carbon mixture growth experiment also revealed that glucose did not cause toxicity or interfere with the metabolism of a gluconeogenic carbon substrate when Mtb was missing both glucokinases.

### PPGK and GLKA can incorporate glucose into Mtb's central carbon metabolism

**Figure 3:**
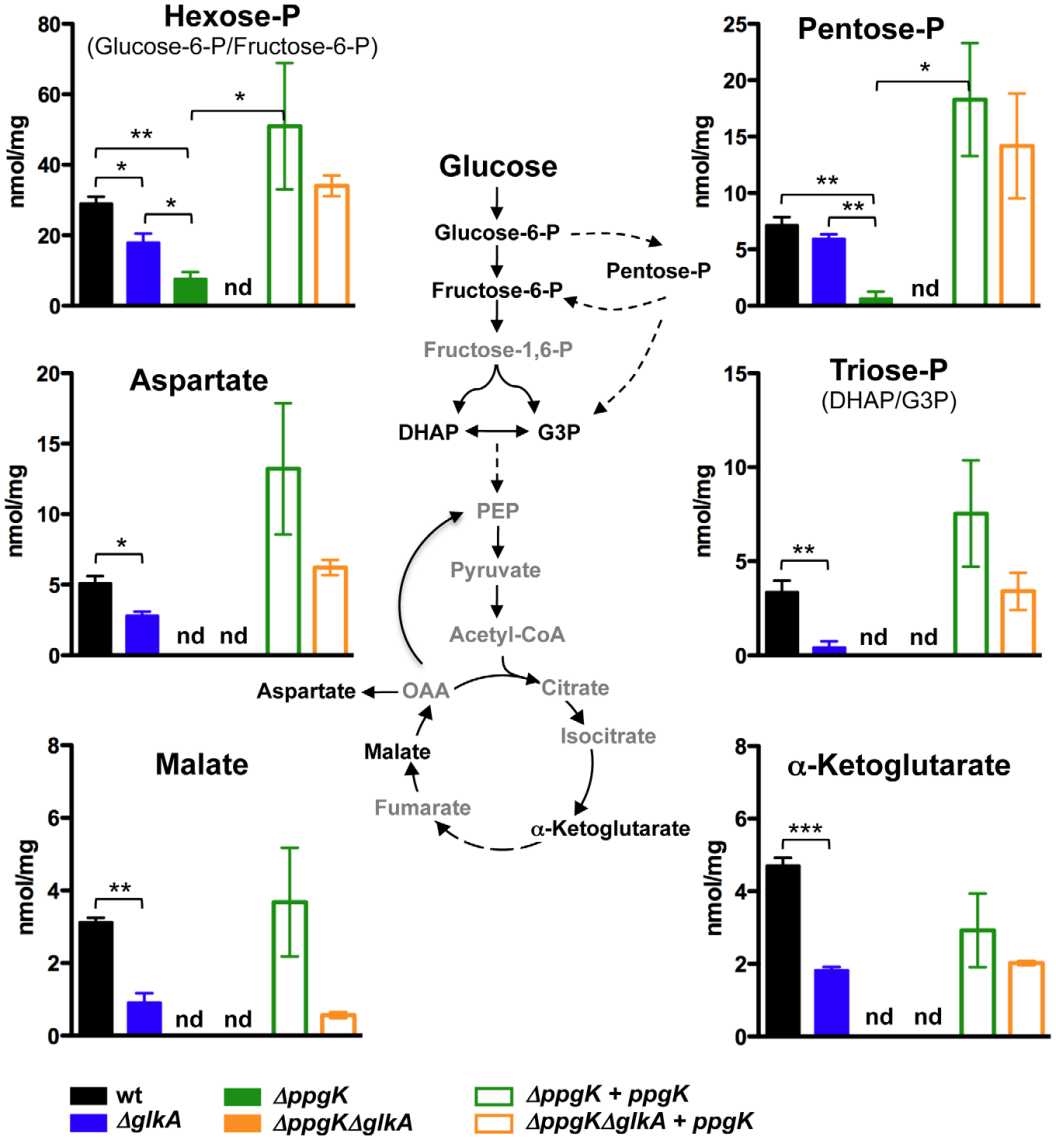
Both PPGK and GLKA mediate glucose incorporation into central carbon metabolism. Schematic illustration of the metabolic pathways studied using carbon tracing analysis and isotopic incorporation of U^13^C-glucose into the intracellular pool of selected metabolites. Isotopic labeling is indicated on the y-axis as nmol labeled/mg protein/16 h labeling interval. Each bar represents the mean of three sample replicates and error bars indicate standard deviation from the mean. nd = not detected. * P≤0.05, ** P≤0.01, *** P≤0.001. Data are representative of two independent experiments.

We studied the incorporation of glucose-derived carbon into central carbon metabolism to gain further insights into Mtb's glucokinases. We examined uniformly-(U)^13^C-labled glucose incorporation into metabolites representative of glycolysis (hexose-6-phosphate (P) and triose-P), pentose phosphate pathway (pentose-P) and the tricaboxylic acid (TCA) cycle (aspartate, malate and α-ketoglutarate). As previously described [16], wt Mtb incorporated U-^13^C-glucose-derived carbons into all the examined central carbon metabolism intermediates (Figure 3). Incorporation of U-^13^C glucose was abolished in Δ*ppgK*Δ*glkA* reflected by the lack of detectable incorporation of U-^13^C glucose-derived carbon into any of the examined central carbon metabolism intermediates (Figure 3). Expression of *ppgK* restored glucose metabolism in Δ*ppgK*Δ*glkA*. This confirmed that Mtb depends on PPGK and GLKA and has no alternative metabolic route for glucose assimilation.

We examined the contribution of each glucokinase ortholog to glucose incorporation. Metabolism of U-^13^C glucose into intermediates of central carbon metabolism was reduced but not abolished in Δ*glkA* compared to wt (Figure 3). We observed a more pronounced decrease of U-^13^C glucose incorporation when Mtb was missing PPGK (Figure 3). Incorporation of U-^13^C-glucose-derived carbon into intermediates of glycolysis (hexose-P and triose-P) and the pentose phosphate pathway (pentose-P) was significantly reduced in Δ*ppgk* and we could not detect ^13^C incorporation into the TCA cycle metabolites α-ketoglutarate, malate or aspartate (Figure 3). Enzymatic quantification confirmed that both glucose-6-P and fructose-6-P pools were reduced in Δ*ppgk* after incubation with glucose as single carbon source (Table S1). Together, these data demonstrate that GLKA is enzymatically competent to phosphorylate glucose, but this activity is not sufficient to support efficient replication on glucose in the absence of PPGK. Hence, GLKA activity cannot meet the cell's demand for energy and biomass production when glucose is the sole carbon source. In conclusion, both PPGK and GLKA can phosphorylate glucose, but PPGK has a predominant role in metabolizing glucose.

### The glucokinase double mutant is attenuated during the chronic phase of infection in mice

**Figure 4:**
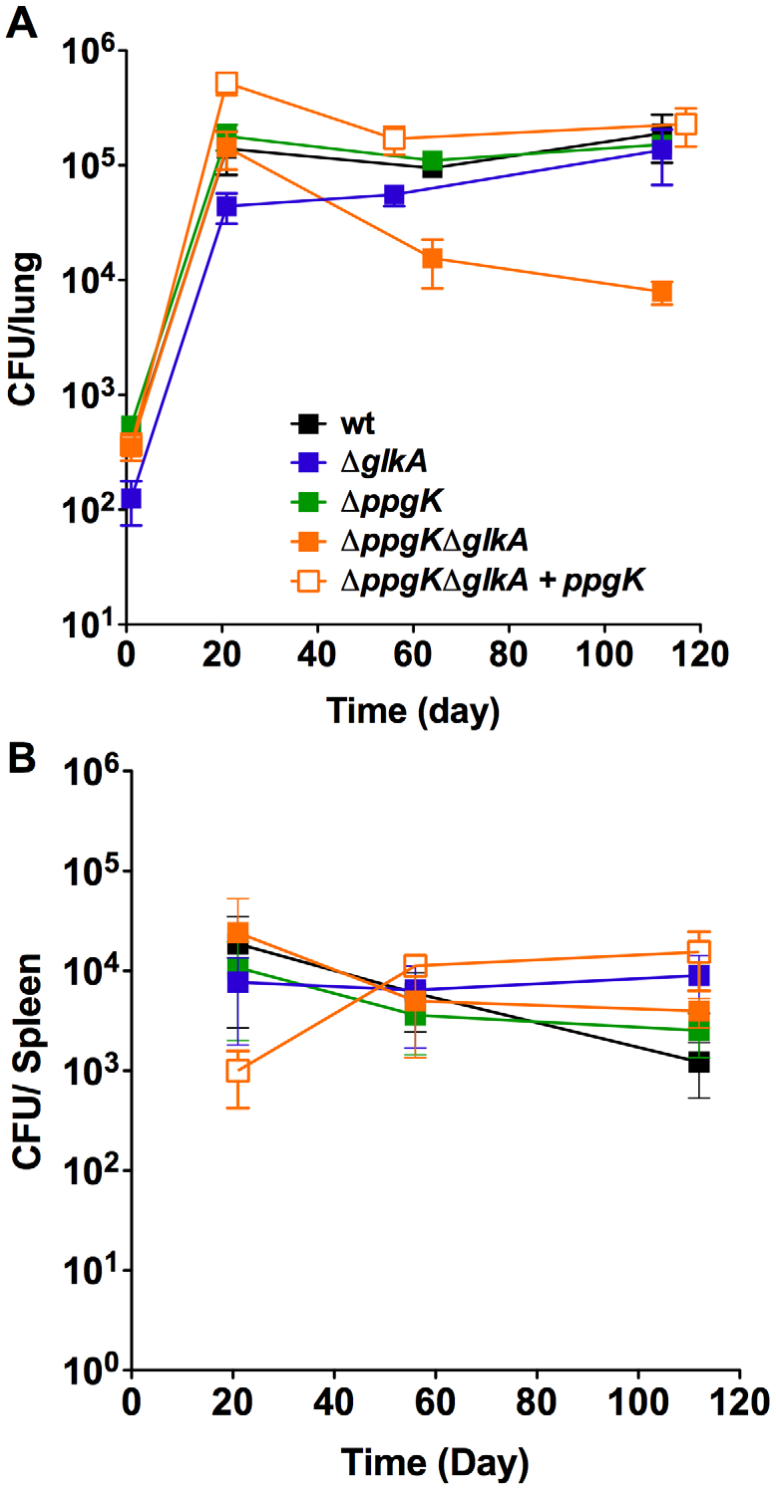
Glucose phosphorylation is required for mycobacterial persistence in lungs of chronically infected mice. Bacterial titers in lungs (A) and spleens (B) from mice infected with wt, glucokinase mutants and complemented mutant. Data are means ± s.d. from four mice per time point per group and represent two independent experiments.

It is unknown whether Mtb has access and metabolizes glucose during growth or persistence in mice and the glucokinase mutants allowed investigating this question. Following low dose aerosol infection Δ*ppgK*Δ*glkA* replicated and established a bacterial load in mice lungs similar to wt Mtb (Figure 4A). However, between days 21 and 56 the Δ*ppgK*Δ*glkA* titer in the lung decreased 1.0 log and further declined 0.25 log between days 56 and 112, indicating that in the absence of PPGK and GLKA Mtb was unable to adapt like wt Mtb to the changes in the host environment associated with the onset of adaptive immunity. Restoring *ppgK* expression complemented the persistence defect of Δ*ppgK*Δ*glkA*. Deletion of either GLKA or PPGK did not impact growth or survival of Mtb in the mouse suggesting that the enzymes can compensate for each other to maintain Mtb fitness during the chronic phase of infection. None of the glucokinase mutants were attenuated in mouse spleens (Figure 4B). These observations revealed that Mtb relies on glucose phosphorylation for normal persistence in mouse lungs but not spleens.

### Loss of glucokinase activity does not impair trehalose metabolism or survival during nutrient starvation

**Figure 5:**
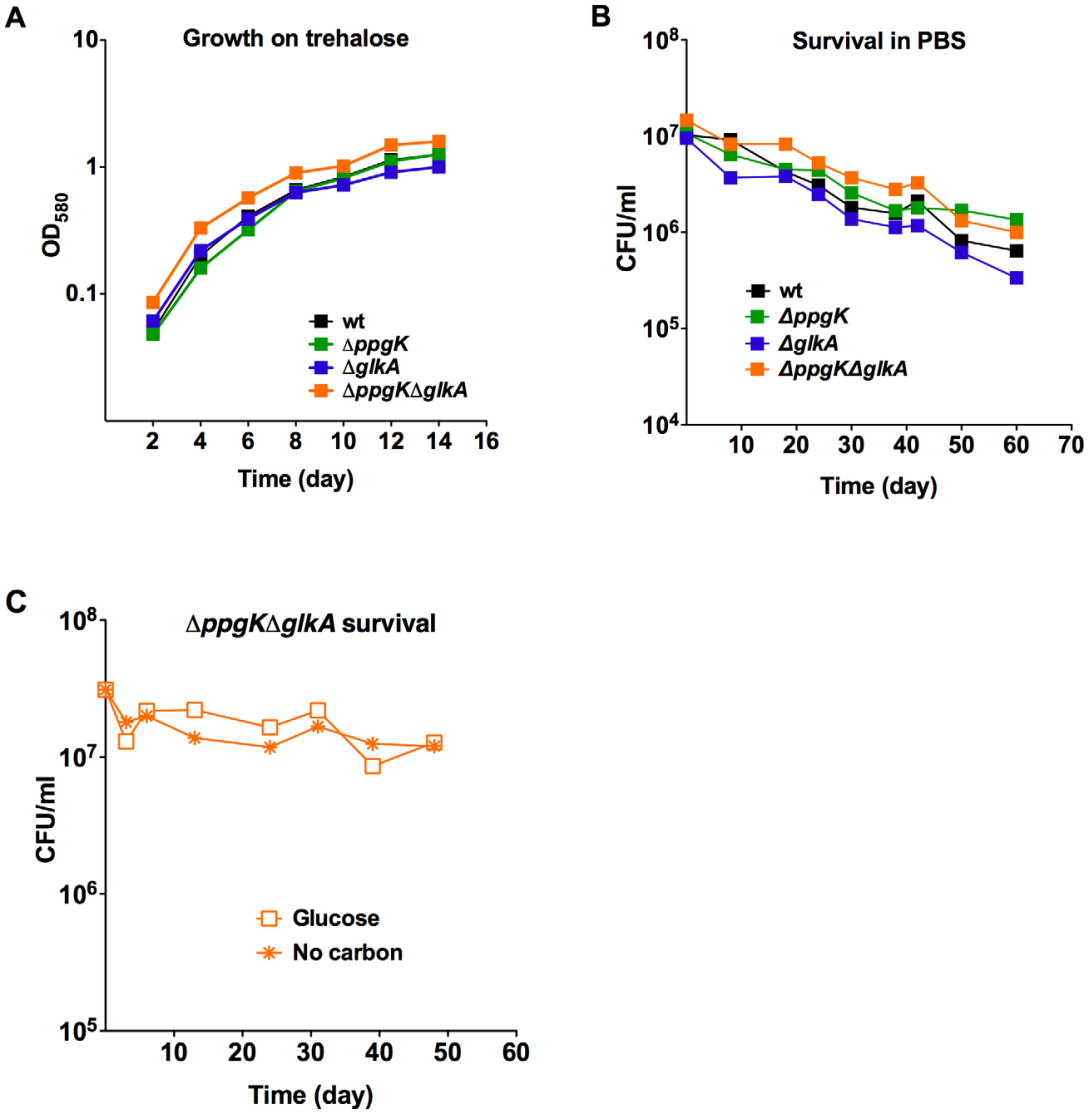
Gluokinases are dispensable for trehalose metabolism and survival during starvation. (A) Growth in carbon defined media with 0.4% trehalose. (B) Survival of wt, *ΔglkA*, *ΔppgK*, and *ΔppgKΔglkA* in PBS. (C) Survival of *ΔppgKΔglkA* in carbon defined media containing 0.4% glucose or no carbon.

Retrograde transport of trehalose was shown to be necessary for Mtb virulence in mice [12]. The authors speculated that trehalose transport could fulfill several metabolic needs including supplying Mtb with a carbon source for growth. The disaccharide trehalose consists of two glucose units and can be converted to glucose by trehalase [17]. We tested whether loss of PPGK and GLKA impacted Mtb's ability to utilize trehalose for growth *in vitro* (Figure 5A). The single and double glucokinase mutants replicated like wt Mtb using trehalose as the sole carbon source demonstrating that Mtb metabolizes trehalose without the need for glucokinase activity. Thus, attenuation of the glucokinase double mutant *in vivo* was not due to an inability to utilize trehalose as a carbon source.

Mtb lacking the stringent response enzyme Rel_Mtb_ was unable to survive prolonged periods of starvation *in vitro* and was attenuated in the chronic phase of infection in mice and guinea pigs [18]–[20]. This work highlighted that adaptation to starvation is an important aspect of Mtb's pathogenicity. We therefore investigated whether loss of glucokinase activity impaired Mtb's ability to adapt to conditions of nutrient starvation and determined whether the glucokinase double mutant was able to survive 60 days of incubation in nutrient free phosphate buffered saline (PBS). Survival of Δ*ppgK*Δ*glkA* in this starvation medium was indistinguishable from wt (Figure 5B) indicating that Mtb's ability to survive nutrient starvation does not depend on glucokinase activity. We also tested whether glucose caused toxicity in Δ*ppgK*Δ*glkA*, which cannot phosphorylate glucose. The glucokinase double mutant was fully viable during prolonged incubation in medium containing glucose as the sole carbon source (Figure 5C). Taken together these experiments revealed that Mtb does not employ glucose metabolism to adapt to starvation.

### Increased susceptibility to oxidative stress is not responsible for the persistence defect of the glucokinase double mutant

**Figure 6:**
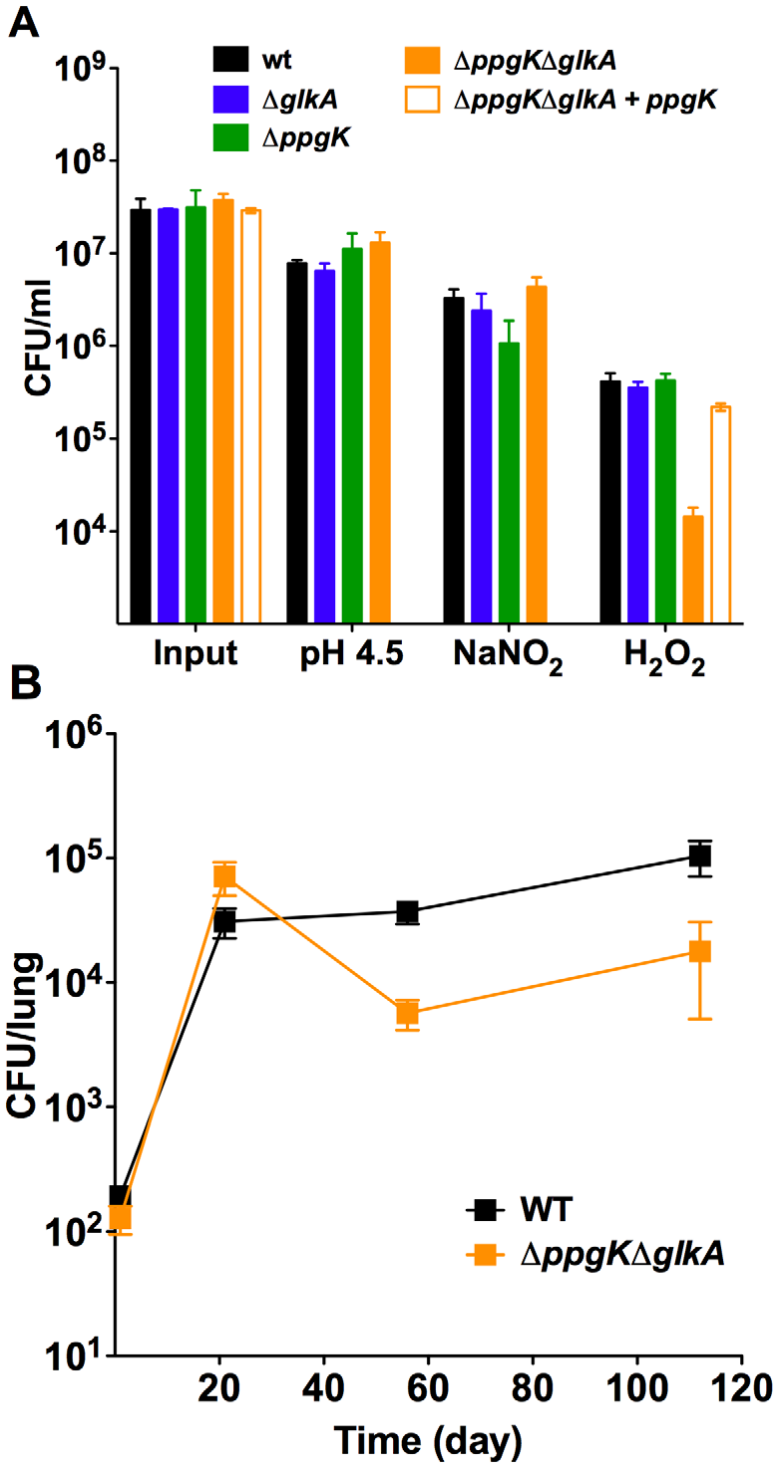
The glucokinase double mutant is hypersusceptible to hydrogen peroxide, but attenuated in phagocyte oxidase deficient mice. (A) Susceptibility to low pH, reactive nitrogen intermediates and hydrogen peroxide. Strains were exposed to pH 4.5 for 6 days, to 3 mM NaNO_2_ at pH 5.5 for 3 days and to 5 mM H_2_O_2_ for 4 hrs and bacterial survival was determined by plating CFU. Data are means ± s.d. of triplicate cultures. Hypersusceptibility of *ΔppgKΔglkA* to 5 mM H_2_O_2_ was demonstrated in three independent experiments. (B) Bacterial titers in lungs of gp96^phox−/−^ mice infected with wt and *ΔppgKΔglkA*. Data are means ± s.d. from three mice per time point per group and representative of two independent experiments.

Mtb persistence in chronically infected mice is achieved through resistance to a diverse array of stresses imposed by the host adaptive immune response [21], [22]. Glucose-6-P has been implicated in resistance of *M. smegmatis* to oxidative stress potentially by serving as a source of reducing power [23]. We investigated whether the Mtb glucokinase mutants were impaired in their ability to resist stresses encountered in the host, such as reactive oxygen species, reactive nitrogen species and low pH. The glucokinase mutants survived similar to wt after exposure to low pH and reactive nitrogen species (Figure 6A). However, Mtb lacking both glucokinases was killed 1 log more than wt after exposure to hydrogen peroxide (H_2_O_2_). This phenotype was fully complemented when *ppgK* expression was restored using an integrative plasmid indicating that the impaired survival after exposure to H_2_O_2_ was solely due to loss of glucokinase activity. This hypersusceptibility was not due inability to induce expression of genes involved resistance to oxidative stress (Table S2). Moreover, KatG and SodA protein amounts were similar in wt and Δ*ppgK*Δ*glkA* (Figure S4). Resistance to H_2_O_2_ mediated oxidative stress was previously shown to be important for Mtb virulence during the chronic phase of infection. Mtb lacking catalase KatG, was hypersuceptible to H_2_O_2_ and attenuated specifically in the chronic phase of infection [24]. *In vivo* attenuation was rescued in mice lacking NADPH oxidase (gp91^Phox−/−^). Thus, removal of oxidative species production by the host NADPH oxidase bypassed the need of catalase for Mtb pathogenesis. To determine if attenuation of Δ*ppgK*Δ*glkA* was dependent on the host respiratory burst we infected gp91^Phox−/−^ mice. Similar to the phenotype in wt mice, viability of Δ*ppgK*Δ*glkA* decreased by 1 log between days 21 and 56 in gp91^Phox−/−^ mouse lungs (Figure 6B) indicating that phagocyte oxidase is dispensable for killing Δ*ppgK*Δ*glkA*. These data suggest that the *in vitro* observed increased susceptibility to H_2_O_2_ does not contribute to the attenuation of Mtb lacking glucokinases pointing to a metabolic requirement for glucose-6-P during survival in chronically infected mice.

## Discussion

That Mtb can metabolize glucose to support its growth in liquid culture has been established over 80 years ago [25]. Whether glucose metabolism is important for the generation of biomass during *in vivo* growth or required during chronic infections was unknown. Here, we investigated the role of Mtb's glucokinases catalyzing the generation of glucose-6-P, the first step in glycolysis and essential for glucose metabolism. We found that Mtb expresses two functional glucokinase ortholgues PPGK and Rv0650, which we named GLKA consistent with the *M. smegmatis* homolog [15]. Protein sequence analysis revealed that PPGK and GLKA belong to the ROK (repressor, ORF, kinase) family, which includes glucokinases and transcriptional repressors [26]. Bioinformatic analysis did not identify a canonical helix-turn-helix motif in PPGK or GLKA commonly found at the amino terminus of transcriptional repressors of the ROK family, suggesting that they do not act as transcriptional regulators. PPGK and GLKA have, however, conserved glucokinase features including the catalytic active aspartate residue, ATP binding domain, and glucose binding sites (Figure S2) [27]. PPGK contains five glucose binding sites and two ATP binding domains spanning amino acid residues 22–31 and 114–156 [13]. Similar to GLKA from *M. smegmatis*, Mtb GLKA contains only three of the five amino acid residues involved in glucose binding and only a single ATP binding domain spanning amino acids 4–17 [15]. This might explain why this enzyme is less efficient than PPGK in incorporating glucose into Mtb's central carbon metabolism and not sufficient to sustain robust growth on glucose in the absence of PPGK. Mtb lacking both PPGK and GLKA glucokinases could not metabolize glucose, which established that Mtb does not have any alternative pathway for glucose assimilation under the conditions tested.

Mtb has been shown to preferentially use host lipids as carbon sources for growth and persistence during infection [2], [4], [5], [9], [10]. In agreement with this, Δ*ppgK*, while severely compromised for growth with glucose as sole carbon source *in vitro*, replicated and survived like wt Mtb in mice. Surprisingly, Mtb lacking both glucokinases PPGK and GLKA lost viability and failed to persist normally during the chronic phase of infection. This revealed that glucose phosphorylation is important for Mtb's fitness in chronically infected mice. The most pronounced decline in viability was observed between week three and eight post infection, while titers declined more slowly later on. This suggests that glucokinase activity helps Mtb adapt to changes in the host environment associated with the onset of adaptive immunity. The *in vivo* attenuation was tissue specific and only observed in lungs but not in spleens. This was reproducible in four independent mouse infections, two infections of wt and two of gp91^phox−/−^ mice. Knowledge regarding differences with respect to the microenvironments Mtb is exposed to in lungs versus spleens is scarce, however it is conceivable that the metabolic constraints in the lung are different from those in the spleen especially following immune activation. The identification and characterization of mutants displaying tissue specific attenuation might help investigate this.

We assessed if the persistence defect was associated with increased susceptibility of Δ*ppgK*Δ*glkA* to stresses Mtb is likely exposed to in response to immune activation of the host [22]. Maintenance of a high glucose-6-P pool was proposed to be important for *M. smegmatis* ability to resist superoxide stress by serving as an electron store for detoxifying the superoxide generating agents plumbagin and menadione [23]. While Δ*ppgK*Δ*glkA* was hypersusceptible to hydrogen peroxide, its attenuation was not rescued in NADPH oxidase deficient mice arguing against the hypothesis that increased susceptibility to oxidative stress caused the *in vivo* phenotype. Δ*ppgK*Δ*glkA* was not hypersusceptible to plumbagin or menadione (data not shown) demonstrating that loss of glucokinases does not sensitize Mtb to all forms of oxidative stress. The reason for the specific hypersusceptibility to hydrogen peroxide is unclear.

We cannot exclude that Mtb's glucokinases phosphorylate an alternative substrate. Broad-specificity hexokinases have mostly been identified in Archea and eukaryotes and are less common in eubacteria [28]–[30]. The hexokinase RokA from *Bacteroides fragilis* has been shown to phosphorylate several substrates including glucose, mannose and *N*-acetyl-D-glucosamine (NAG) [30]. This unusual NAG kinase may act as activator for the synthesis of capsule components and be important for recycling of cell wall material [30]. To attribute the *in vivo* attenuation of Δ*ppgK*Δ*glkA* to loss of NAG kinase activity, both enzymes would need to be able to phosphorylate NAG, which seems unlikely, given the high affinity of PPGK for glucose [14]. Furthermore, none of the glucokinase mutants exhibited increased susceptibility to the antibiotic cycloserine, which targets peptidoglycan biosynthesis [31], [32] or to the detergent SDS (data not shown). Thus, we did not find evidence that deletion of *ppgK* and *glkA* affected permeability of the mycobacterial cell envelope.

Glucose-6-P has many important cellular functions such as energy production through glycolysis, production of reducing power through the pentose phosphate pathway, and it serves as an important precursor for anabolic processes, especially cell wall and nucleotide biosynthesis. Gluconeogenesis could potentially produce the necessary glucose-6-P to meet cellular demands. However, glucose-6-P generation directly from glucose via glucokinase consumes one molecule of ATP while its production through gluconeogenesis is more energetically costly. Exposure to diverse stresses associated with the host immune response might compromise ATP production in Mtb through the electron transport chain, such that ATP production through substrate level phosphorylation using glucose-6-P as a precursor might become important.

The glucokinase mutants replicated normally with trehalose as sole carbon source indicating that trehalose metabolism does not depend on trehalase. Trehalase hydrolyzes trehalose into two molecules of glucose, while trehalose phosphorylase catalyzes the phosphorolytic cleavage of trehalose into glucose1-P and glucose [33]. A phosphate dependent trehalase has been purified from *M. smegmatis* and Mtb contains a homolog [17]. Despite the requirement for phosphate, there was no evidence of phosphorolytic cleavage, suggesting that the enzyme produces two molecules of glucose, which require glucokinase activity for entry into glycolysis. Our data indicate that Mtb is able to bypass glucose phosphorylation when growing on trehalose.

How Mtb takes up glucose remains to be determined. In contrast to *M. smegmatis* which was predicted to encode 28 carbohydrate transporters, only five such transporters were identified in the Mtb genome [8]. Direct biochemical and structural analyses of these is lacking and glucose transport across the outer membrane of Mtb is even less well understood [34], [35].

Our work identified a previously unrecognized role for glucose phosphorylation in Mtb pathogenesis. Recent work showed that Mtb is metabolically flexible and capable of utilizing multiple carbon substrates simultaneously [16]. The *in vivo* attenuation of Mtb lacking both glucokinases, PPGK and GLKA, suggests that Mtb has access to glucose and relies on glucose phosphorylation to survive during chronic infection. While the physiological function of the generated glucose-6-P remains unclear, the impact of loss of glucokinase activity emphasizes that Mtb's metabolic flexibility is crucial to its pathogenesis and that the pathogen does not exclusively rely on metabolism of host lipids.

## Materials and Methods

### Ethics statement

All procedures including animal studies were conducted following the National Institutes of Health guidelines for housing and care of laboratory animals and performed in accordance with institutional regulations after protocol review and approval by the Institutional Animal Care and Use Committee of Weill Cornell Medical College (protocol # 0601-441A, Conditional Expression of Mycobacterial Genes).

### Strains, media and culture conditions

*M. tuberculosis* (Erdman) strains were routinely grown at 37°C in liquid Middelbrook 7H9 medium (Difco) containing 0.2% glycerol, 0.5% bovine serum albumin, 0.2% dextrose, 0.085% NaCl, and 0.05% Tween 80 or on Middlebrook 7H10 agar plates containing 10% OADC supplement (Becton Dickinson) and 0.5% glycerol. For growth in carbon defined media, we used 7H9 with 0.05% Tyloxapol and a carbon substrate at the indicated concentration (% wt/vol). When required, hygromycin B was used at a concentration of 50 µg/ml and kanamycin at a concentration of 25 µg/ml.

### Mutant strain and plasmid constructions

Mtb knockout strains were constructed via allelic exchange using the conditionally replicating mycobacteriophage method as described elsewhere [5], [36]. The double knockout glucokinase strain was constructed by first removing the hygromycin cassette from the Δ*ppgK* genome by expressing the Cre recombinase from an unstable plasmid. The *glkA* gene was subsequently deleted in the Δ*ppgK* strain that was cured of both the hygromycing cassette and the Cre expression plasmid. All Mtb knockout strains were confirmed using Southern Blot Analysis. Complementation plasmids were constructed using the Gateway Cloning Technology (Invitrogen) as described previously [37]. For complementation the *ppgK* gene was cloned containing its putative promoter (350 nucleotides upstream of the start codon) into a plasmid that integrates into the chromosomal attB site. To complement Δ*ppgK* with *glkA*, the *glkA* gene was cloned into an episomal plasmid behind the strong mycobacterial promoter P*smyc* [38]. Primer sequences are available upon request.

### Liquid chromatography-mass spectrometry

Metabolites were separated on a Cogent Diamond Hydride Type C column (Microsolve Technologies). The mass spectrometer used was Agilent Accurate Mass 6220 TOF coupled to an Agilent 1200 LC system. Dynamic mass axis calibration was achieved by continuous infusion of a reference mass solution using an isocratic pump with a 100∶1 splitter. This configuration achieved mass errors of approximately 5 parts-per-million (ppm), mass resolution ranging from 10,000–25,000 (over m/z 121–955 amu), and 5 log_10_ dynamic range. Detected ions were deemed metabolites on the basis of unique accurate mass-retention time (AMRT) identifiers for masses exhibiting the expected distribution of accompanying isotopomers. Metabolite identities were established by querying against a pre-populated AMRT library of metabolite standards and demonstrating chromatographic co-elution of candidate metabolites with pure chemical standards spiked into representative biological samples.

### Isotopomer data analysis

The extent of isotopic labeling for each metabolite was determined by dividing the summed peak height ion intensities of all labeled species by the ion intensity of both labeled and unlabeled species, expressed in percent. Label-specific ion counts were corrected for naturally occurring ^13^C species (i. e. [M+1] and [M+2]). The relative abundance of each isotopically labeled species was determined by dividing the peak height ion intensity of each isotopic form (corrected for naturally occurring ^13^C species as above) by the summed peak height ion intensity of all labeled species.

### Mouse infections

Female 7–8 week old C57BL/6 and gp91^phox−/−^ mice (Jackson Laboratory) were infected with *Mtb* by aerosol as described [39]. Bacterial numbers were determined by plating homogenized organs for CFU.

### Stress assays

Starvation survival experiments were carried by inoculating ∼5×10^6^ bacteria in PBS+0.5% Tyloxapol or in Sauton's base medium (0.5 g/L KH_2_PO_4_, 0.5 g/L MgSO_4_, 2.0 g/L citric acid, 0.05 g ferric ammonium citrate, 0.5 g/L ammonium sulfate, 0.05% Tyloxapol, pH 7.4) supplemented with 0.4% glucose or no carbon. Bacterial survival in these media was determined by periodically plating serial dilutions on 7H10 plates to estimate CFU/ml. Susceptibility to nitrosative, oxidative stress and low pH was determined as described previously [39], [40].

## Supporting Information

Figure S1 **Southern blot analysis of glucokinase mutants.** (A) Schematic representation of the *ppgK* genomic region in wt, *ΔppgK* and *ΔppgKΔglkA*, in which the hygromycin cassette was removed. (B) Southern blot of chromosomal DNA digested with *Pvu*I+*Pvu*II from wt (lane1), *ΔppgK* (lane 2) and *ΔppgKΔglkA* (lane 3). (C) Schematic representation of *glkA* genomic region in wt and ΔglkA. (D) Southern blot of chromosomal DNA digested with SacI from wt (lane 1), *ΔglkA* (lane 2) and *ΔppgKΔglkA* (lane 3).
(PDF)

Figure S2 **Comparison of Mtb's glucokinases, GLKA and PPGK.** (A) Amino acid sequence alignment of PPGK and GLKA. The overall sequence identity is 22%. (B) Schematic representation of PPGK and GLKA. Residues involved in glucose binding are indicated in red with corresponding residue number shown below. Grey boxes represent ATP binding domains and the catalytic aspartate residues are marked with an asterisk.
(PDF)

Figure S3 **Quantification of** ***ppgK*** **and** ***glkA*** **(** ***Rv0650*** **) transcript levels in wt Mtb and mutants.** Absolute mRNA amounts were determined by quantitative real time PCR. Serial dilutions of defined copy numbers of the Mtb chromosome were included for each real-time PCR to generate standard curves, which were used to calculate the absolute copy number of each gene. Data are means from 3 independent replicates ± SD.
(TIFF)

Figure S4 **KatG and SodA amounts in cell extracts.** KatG and SodA were detected by immunoblotting in lysates from wt, *ΔppgK, ΔglkA* and *ΔppgKΔglkA*.
(TIFF)

Table S1 **Enzymatic quantification of glucose-6-P and fructose-6-P metabolite pools in cell extracts from wt and glucokinase mutants.** Metabolite amounts were measured as described in Materials and Methods. Data represent the mean of three independent replicates from two independent experiments ± SEM. * P≤0.05 compared to wt.
(PDF)

Table S2 **Expression of antioxidant genes in wt and** ***ΔppgKΔglkA*** **.** mRNA amounts were quantified by quantitative real time PCR before and after exposure to 5 mM H_2_O_2_ for 30 min and normalized to *sigA*. Data represent the mean of three experimental replicates ± SD.
(PDF)
