# Supplementary figures and images for: Glucose Phosphorylation Is Required for Mycobacterium tuberculosis Persistence in Mice
Source: PLoS Pathog. 2013 Jan 10;9(1):e1003116. doi: 10.1371/journal.ppat.1003116 (PMC3542180; doi:10.1371/journal.ppat.1003116)

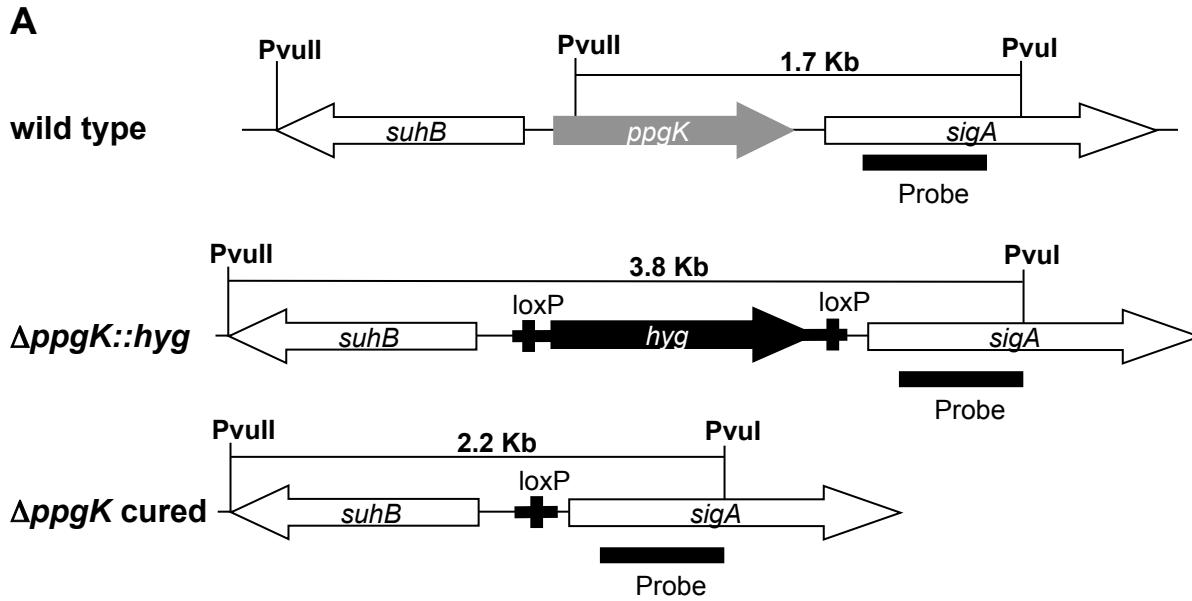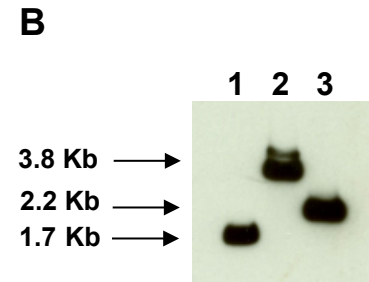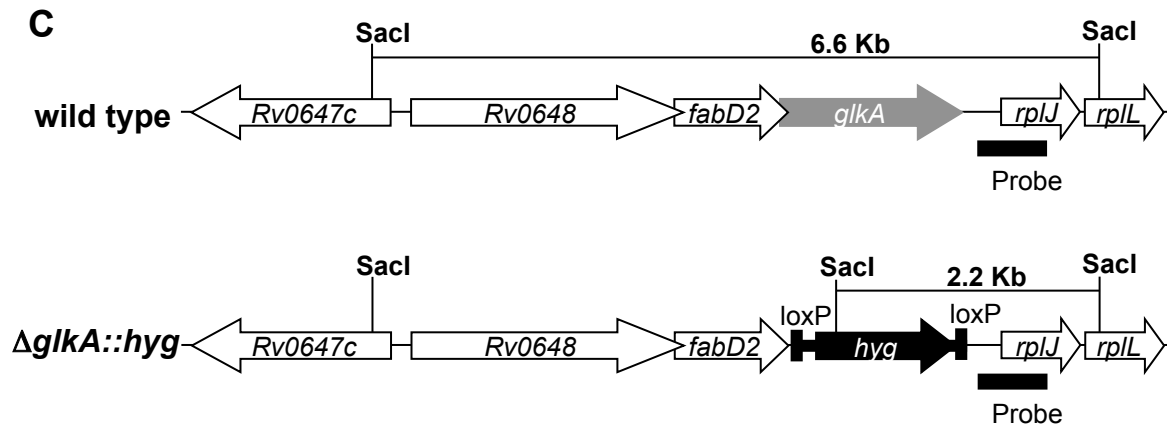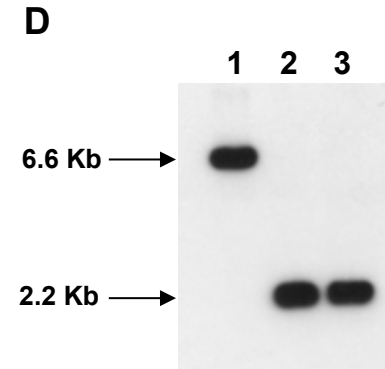

Supplement: Figure S1 — Southern blot analysis of glucokinase mutants. (A) Schematic representation of the ppgK genomic region in wt, ΔppgK and ΔppgKΔglkA, in which the hygromycin cassette was removed. (B) Southern blot of chromosomal DNA digested with PvuI+PvuII from wt (lane1), ΔppgK (lane 2) and ΔppgKΔglkA (lane 3). (C) Schematic representation of glkA genomic region in wt and ΔglkA. (D) Southern blot of chromosomal DNA digested with SacI from wt (lane 1), ΔglkA (lane 2) and ΔppgKΔglkA (lane 3). (PDF) [file ppat.1003116.s001.pdf]

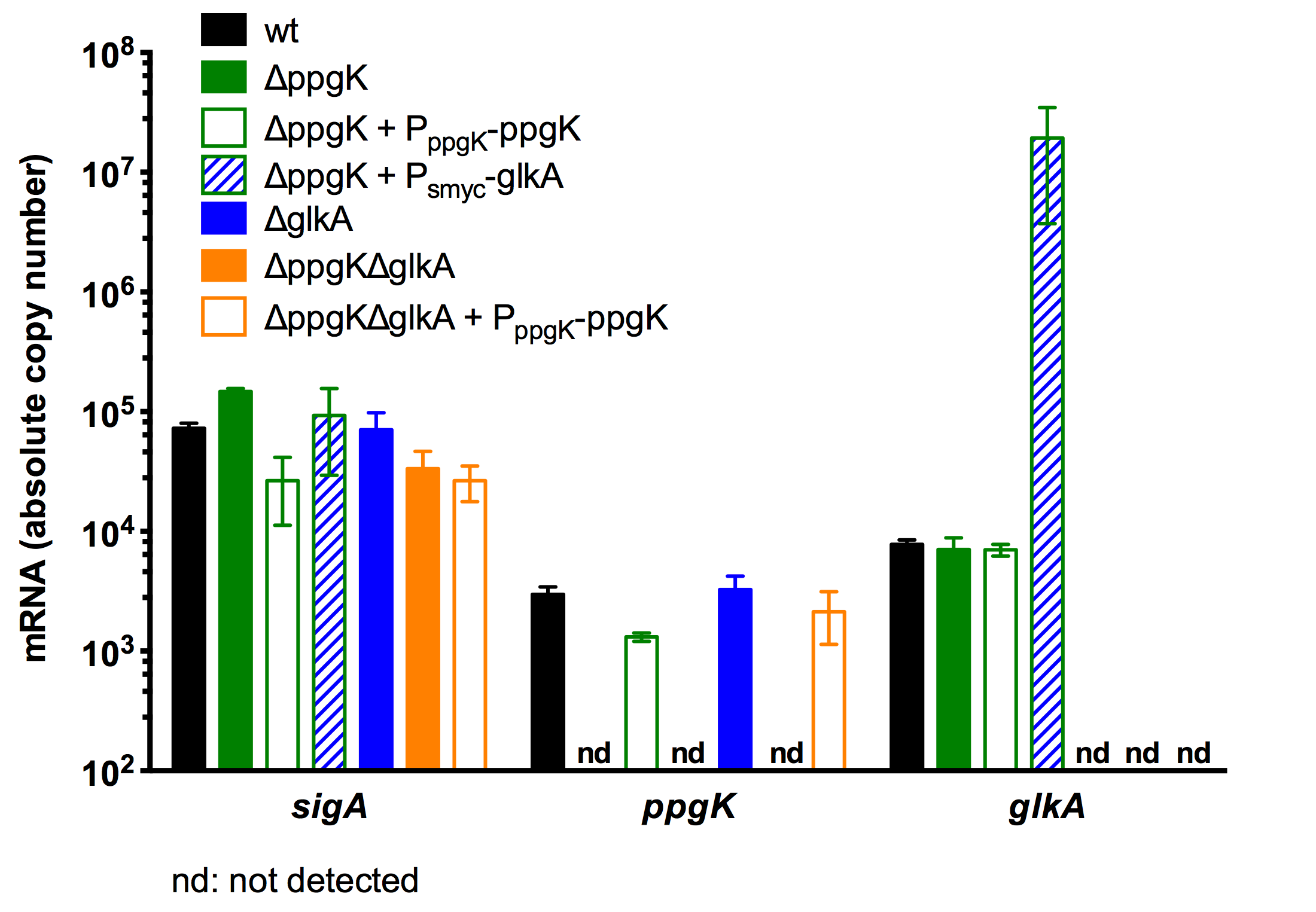

Supplement: Figure S3 — Quantification of ppgK and glkA ( Rv0650 ) transcript levels in wt Mtb and mutants. Absolute mRNA amounts were determined by quantitative real time PCR. Serial dilutions of defined copy numbers of the Mtb chromosome were included for each real-time PCR to generate standard curves, which were used to calculate the absolute copy number of each gene. Data are means from 3 independent replicates ± SD. (TIFF) [file ppat.1003116.s003.tiff]

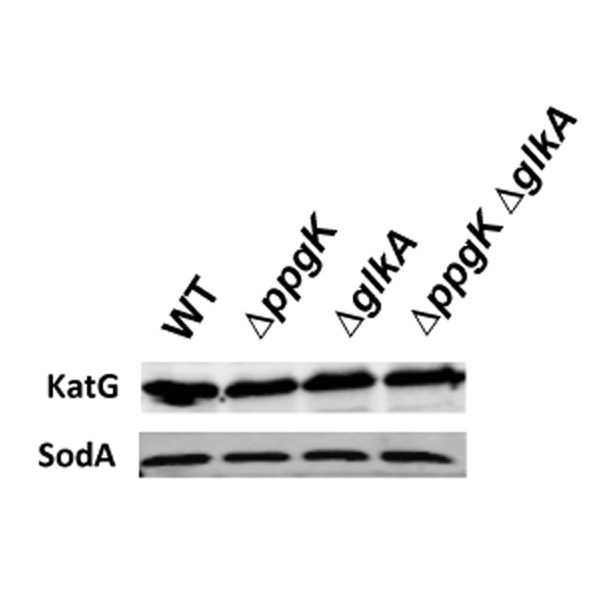

Supplement: Figure S4 — KatG and SodA amounts in cell extracts. KatG and SodA were detected by immunoblotting in lysates from wt, ΔppgK, ΔglkA and ΔppgKΔglkA. (TIFF) [file ppat.1003116.s004.tiff]
