# Supplementary material for: Glucose Phosphorylation Is Required for Mycobacterium tuberculosis Persistence in Mice
Source: PLoS Pathog. 2013 Jan 10;9(1):e1003116. doi: 10.1371/journal.ppat.1003116 (PMC3542180; doi:10.1371/journal.ppat.1003116)

A

```
CLUSTAL W (1.83) multiple sequence alignment


PPGK            MTSTGPETSETPGATTQRHGFGIDVGGSGIKGGIVDLDTGQLIGDR--IKLLTPQPATPL
GLKA            -----------------MLTLCLDIGGTKIAAGLADPAGTLVHTAQRPTPAYGGAEQVWA
                                    : :*:**: * .*:.*     :   :           .

PPGK            AVAKTIAEVVNGFG-WRGPLGVTYPGVVTHGVVRTAANVDKSWIGTNARDTIGAELGGQQ
GLKA            AVAEMIADALGVAGGAVGGVGIASAGPIDLHSGRVSPINIGSWGGFPLRDRVAAAVPGVP
                ***: **:.:.  *   * :*:: .* :     *.:.    ** *   ** :.* : *

PPGK            VTILNDADAAGLAETRYGAGKNNPGLVVLLTFGTGIGSAVIHN--------------GTL
GLKA            VRLGGDGVCMALGEHWLGAGRG-ARFLLGLVVSTGVGGGLVLDGAPCLGRTGNAGHVGHV
                * : .*. . .*.*   ***:. . ::: *...**:*..:: :              * :

PPGK            IPNTEFGHLEVGGKEAEERAASS-----VKEKNDWTYP----------------------
GLKA            VVDPDGSPCPCGGRGCVETIASGPSLARWARANGWSAPPGAGAKELAEAAGAGDPVALRA
                : :.: .    **: . *  **.       . *.*: *

PPGK            --KWAKQVIRVLIAIENAIWPDLFIAGGGISR------------KADKWVPLLENRTPVV
GLKA            FRRGAAALAAMIASVGAVCDLDLAVIGGGVAKSGRLLFEPLRAALADHARLDFLAGLRVV
                  : *  :  :: ::  .   ** : ***:::             **:    :     **

PPGK            PAALQNTAGIVGAAMASVADTTH
GLKA            PAELGGAAGLVGAARLAAIA---
                ** * .:**:****  :.
```

B

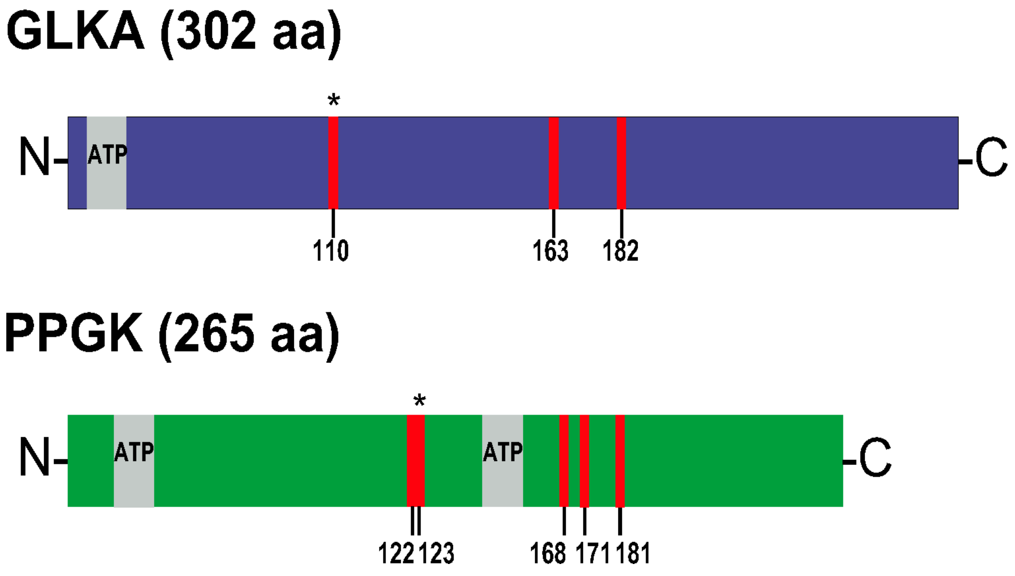

Supplement: Figure S2 — Comparison of Mtb's glucokinases, GLKA and PPGK. (A) Amino acid sequence alignment of PPGK and GLKA. The overall sequence identity is 22%. (B) Schematic representation of PPGK and GLKA. Residues involved in glucose binding are indicated in red with corresponding residue number shown below. Grey boxes represent ATP binding domains and the catalytic aspartate residues are marked with an asterisk. (PDF) [file ppat.1003116.s002.pdf]
