# Supplementary material for: Glucose Phosphorylation Is Required for Mycobacterium tuberculosis Persistence in Mice
Source: PLoS Pathog. 2013 Jan 10;9(1):e1003116. doi: 10.1371/journal.ppat.1003116 (PMC3542180; doi:10.1371/journal.ppat.1003116)
Supplement: Table S1 — Enzymatic quantification of glucose-6-P and fructose-6-P metabolite pools in cell extracts from wt and glucokinase mutants. Metabolite amounts were measured as described in Materials and Methods. Data represent the mean of three independent replicates from two independent experiments ± SEM. * P≤0.05 compared to wt. (PDF) [file ppat.1003116.s005.pdf]

Table S1. Enzymatic quantification of of glucose-6-P and fructose-6-P metabolite pools in cell extracts from wt and glucokinase mutants.

**Strain**	Grown with glucose		Grown with glycerol	
	**Glucose-6-P** (pmol/mg)	**Fructose-6-P** (pmol/mg)	**Glucose-6-P** (pmol/mg)	**Fructose-6-P** (pmol/mg)
wt	5408 ± 956	2713 ± 554	1860 ± 197	1614 ± 74
Δ*ppgK*	1351 ± 526 *	1365 ± 484 *	1617 ± 375	1576 ± 819
Δ*glkA*	4386 ± 1765	2075 ± 1154	1916 ± 104	1709 ± 839
Δ*ppgK*Δ*glkA*	nd	nd	1807 ± 529	1706 ± 901
Δ*ppgK* + *ppgK*	4402 ± 1520	2728 ± 920	1567 ± 310	1542 ± 715
Δ*ppgK*Δ*glkA* + *ppgK*	5456 ± 2922	3029 ± 1341	2610 ± 819	1971 ± 623
