# Supplementary material for: Glucose Phosphorylation Is Required for Mycobacterium tuberculosis Persistence in Mice
Source: PLoS Pathog. 2013 Jan 10;9(1):e1003116. doi: 10.1371/journal.ppat.1003116 (PMC3542180; doi:10.1371/journal.ppat.1003116)
Supplement: Table S2 — Expression of antioxidant genes in wt and ΔppgKΔglkA . mRNA amounts were quantified by quantitative real time PCR before and after exposure to 5 mM H2O2 for 30 min and normalized to sigA. Data represent the mean of three experimental replicates ± SD. (PDF) [file ppat.1003116.s006.pdf]

Table S2. Expression of antioxidant genes in wt and Δ*ppgK*Δ*glkA*.

**Gene**	mRNA fold induction	
	**wt**	**Δ*ppgK*Δ*glkA***
katG	31.8 ± 9.8	53.0 ± 3.5
ahpC	3.0 ± 0.4	3.0 ± 2.1
thiX	2.0 ± 0.4	6.0 ± 0.8
trxB1	19.3 ± 1.7	36.9 ± 11.4
trxB2	6.1 ± 4.4	17.7 ± 4.4
